# Turkish adaptation of the Children’s Numeracy Interest Scale and its relationship with parental math involvement

**DOI:** 10.1186/s40359-026-05423-3

**Published:** 2026-09-01

**Authors:** Hilal Karakuş Özkan

**Affiliations:** Faculty of Education, Department of Preschool Education, Sinop University, Sinop, Türkiye

**Keywords:** Children’s numeracy interest, Parental math involvement, Home numeracy environment, Preschool children, Psychometric validation, Measurement invariance, Structural equation modeling

## Abstract

**Background:**

Children’s numeracy interest is increasingly recognized as an important affective dimension of early mathematical development. However, to the best of my knowledge, no empirical study has directly examined the association between parental math involvement and children’s numeracy interest in Türkiye, and no validated Turkish-language instrument for assessing this construct has been reported. This study aimed to adapt the Children’s Numeracy Interest Scale into Turkish and examine whether parental math involvement predicted children’s numeracy interest.

**Methods:**

The research comprised in two studies. Study 1 focused on the Turkish adaptation of the Children’s Numeracy Interest Scale and evaluated its psychometric properties using exploratory and confirmatory factor analyses. Study 2 examined whether overall parental math involvement and its domains predicted children’s numeracy interest among parents of preschool children. The proposed models were tested using structural equation modeling, with relevant child and family characteristics included as control variables.

**Results:**

The Turkish version of the Children’s Numeracy Interest Scale demonstrated a one-factor structure, good reliability, and acceptable model fit. Parental math involvement was positively associated with children’s numeracy interest and was a significant positive predictor in the structural model. When the domains of parental math involvement were examined simultaneously, only Numbers and Operations emerged as a significant positive predictor. Among the control variables, child age was also a significant positive predictor of children’s numeracy interest.

## Introduction

Early numeracy is widely regarded as an important foundation for children’s subsequent mathematical learning and broader educational development [1, 2]. During the preschool years, children develop a range of early mathematical competencies, including numeracy-related skills such as number knowledge, counting, and early arithmetic operations [3], as well as mathematical language and spatial skills [4]. However, early mathematical development should not be understood solely in terms of cognitive skill acquisition. Children’s interest in numeracy-related activities is also important because it reflects an affective dimension of early numeracy development and may be relevant to later mathematics learning [1]. More broadly, recent systematic reviews and meta-analyses indicate that several affective and motivational factors—including mathematics-related enjoyment [5, 6], perceived value [5], and motivation [7]—are positively associated with mathematical learning and achievement across different developmental stages. In addition, engagement during early childhood has been positively associated with academic achievement more generally [8]. Accordingly, examining children’s numeracy interest offers a broader perspective on early mathematical development by extending attention beyond their mathematics performance alone.

### Parental math involvement and children’s numeracy interest

The home environment plays a central role in children’s early mathematical experiences. Within this context, parental math involvement refers to parents’ engagement in everyday activities and interactions that support children’s mathematical learning. These activities may include counting objects, comparing quantities, using numbers in daily routines, playing number games, engaging in measurement-related activities, and identifying shapes [9–11]. However, parental math involvement should not be conceptualized as a unitary construct, because home mathematics activities comprise multiple dimensions that may show varying associations with children’s mathematical development [4, 12]. Consistent with this view, associations between the home mathematics environment and children’s mathematical development vary according to the type of parental activity and the specific mathematical outcome examined [4, 13]. Thus, relying only on an overall involvement score may not adequately reflect potentially different associations across mathematical domains, supporting the examination of parental math involvement both as an overall construct and at the domain level.

This study integrates the four-phase model of interest development [14] with ecological systems theory [15] to explain the proposed conceptual pathway linking parental math involvement with children’s numeracy interest. The two perspectives offer complementary components of a unified conceptual framework. Ecological systems theory identifies the family microsystem as the immediate developmental context in which children encounter learning experiences through everyday interactions with their parents [15], whereas the four-phase model explains how repeated and supportive experiences within this context may contribute to the development of interest over time [14].

According to the four-phase model, situational interest may initially be triggered when an activity or interaction captures a child’s attention and provides a positive or meaningful experience. When such experiences are repeated and supported, situational interest may be maintained and gradually develop into a more enduring individual interest [14]. Within the family microsystem, parent–child mathematical activities may provide recurring opportunities to direct children’s attention to mathematical content, encourage active participation, and create enjoyable or meaningful numeracy-related experiences. Thus, ecological systems theory specifies the proximal context in which parental math involvement occurs, whereas the four-phase model explains how repeated experiences within this context may trigger and maintain children’s situational interest in numeracy and, over time, contribute to the development of a more enduring interest.

Recent systematic reviews and meta-analyses indicate that the home mathematics environment is multidimensional, encompassing not only the frequency and type of mathematical activities but also affective components such as parental attitudes, beliefs, expectations, and mathematical communication [13, 16]. Accordingly, parental math involvement should not be conceptualized as a single, uniform construct. Different home mathematical experiences may relate to different outcomes in children’s early mathematics learning. For example, longitudinal evidence suggests that both operational numeracy activities and shared number-game play are associated with later arithmetic fluency and growth in number-comparison abilities [17]. Consistent with this multidimensional view, number-related home activities have been found to comprise two distinct dimensions: play-based number activities and the use of number-related educational materials [12].

The dimensions of parental math involvement may differ in their relevance to children’s numeracy interest because they involve different mathematical content. Activities involving Numbers and Operations may be more closely aligned with the number-focused experiences assessed in measures of children’s numeracy interest, including number identification, counting, and number writing [1, 18]. In contrast, activities involving Geometry, Measurement, and Pattern may be more closely related to children’s interest in those specific mathematical domains. From the perspective of the four-phase model of interest development, activities that closely correspond to a particular content area and provide repeated opportunities for engagement may be more likely to trigger and maintain situational interest [14]. Taken together, these considerations support examining parental math involvement at the domain level and provide a rationale for exploring how specific home-based mathematical experiences relate to children’s numeracy interest.

A considerable body of research has examined the association between parental involvement in home-based mathematical activities and children’s early mathematical skills [1, 17, 19–27]. In contrast, research focusing specifically on children’s numeracy interest and its association with parental math involvement remains comparatively limited [1, 18, 28–30]. This gap is important because children’s numeracy interest may reflect children’s motivation, curiosity, and positive engagement in early mathematical activities. Longitudinal evidence has also linked preschool children’s mathematics-related interest to later symbolic and non-symbolic number skills [18]. Accordingly, Cheung et al. [1] emphasized that researchers and practitioners should consider numeracy interest alongside numeracy competence in early numeracy development.

Broader evidence indicates that young children’s mathematics-related interest is relevant to their early mathematical development. Preschool children with greater math-specific interest have been found to demonstrate stronger emergent mathematics skills [31], whereas more frequent participation in numeracy play during the preschool years was associated with greater confidence in mathematics at age 10 and, among children from higher socioeconomic backgrounds, greater liking of mathematics [32]. Although direct evidence remains limited, the available studies provide useful insights into how parental math involvement may be associated with children’s numeracy interest. For example, Cheung et al. [1] found that, among disadvantaged young Filipino children, numeracy interest was associated with parents’ practices and attitudes. Related longitudinal evidence from Hong Kong further showed that mother–child home numeracy activities were associated with preschool children’s mathematics-related interest, measured using items referring to number-focused activities [18]. Extending this evidence, Cheung et al. [29] found that both the frequency of home numeracy activities and parents’ use of indirect numeracy teaching strategies were positively associated with children’s numeracy interest among Hong Kong kindergarteners. Several studies have also suggested that home numeracy activities may mediate the association between parents’ attitudes toward their children’s numeracy learning and children’s numeracy interest [28, 30]. Taken together, these findings suggest that parental attitudes and practices, particularly home numeracy activities, may be relevant to children’s numeracy interest. However, much of the available direct evidence comes from a limited number of research groups and has been generated primarily in East and Southeast Asian contexts, highlighting the need for research in more culturally diverse settings. To the best of my knowledge, no direct empirical study has examined the association between parental math involvement and preschool children’s numeracy interest in Türkiye.

### Measurement of children’s numeracy interest in Türkiye

Measurement is another important consideration. Reliable assessment of children’s numeracy interest across different cultural contexts requires instruments that are both psychometrically robust and culturally appropriate. Recent work has highlighted the need for stronger psychometric evidence for early numeracy measures, particularly regarding their content validity [2]. Although Cheung et al.’s [1] Children’s Numeracy Interest Scale provides a valuable instrument for assessing children’s numeracy interest, its validity and applicability in the Turkish context have not yet been established. Moreover, to the best of my knowledge, no validated Turkish-language version of this scale has been reported for use with preschool children in Türkiye. This apparent measurement gap may limit research on children’s numeracy interest in the Turkish early childhood context and makes cross-cultural comparisons more difficult. Adapting and validating this scale would therefore contribute to early childhood mathematics research by providing a culturally appropriate instrument for assessing this construct in Türkiye.

### Present study

Taken together, the literature points to two related gaps in the Turkish early childhood context: the absence of a validated Turkish-language measure of children’s numeracy interest and the lack of direct empirical evidence on its association with parental math involvement. Addressing these gaps requires both a culturally appropriate instrument and an examination of the factors associated with children’s numeracy interest. Therefore, the present research comprised two complementary studies. Study 1 aimed to adapt the Children’s Numeracy Interest Scale into Turkish and evaluate its psychometric properties, whereas Study 2 examined the associations of overall parental math involvement and its domains with children’s numeracy interest. By addressing these measurement and empirical gaps, the present study contributes to the early childhood mathematics literature by providing a validated Turkish version of the scale and extending knowledge of the associations etween parental math involvement and children’s numeracy interest in Türkiye.

### Study 1

Study 1 aimed to adapt the Children’s Numeracy Interest Scale into Turkish and evaluate its psychometric properties using parent-reported data on preschool children in Türkiye. To this end, the scale was translated and culturally adapted. The psychometric evaluation focused on its structural validity, assessed through exploratory factor analysis (EFA) and confirmatory factor analysis (CFA), as well as internal consistency, and measurement invariance across child gender groups.

### Study 2

Study 2 examined whether overall parental math involvement positively predicted children’s numeracy interest and explored which domains of parental math involvement significantly predicted children’s numeracy interest. To evaluate the robustness of the findings, H1 was tested and RQ1 was addressed first in models without control variables and then in models that included relevant child and family characteristics as control variables.

Relevant child and family characteristics, including child age, child gender, duration of preschool attendance, parental education, and number of siblings, were included as control variables in the structural models. These variables were not treated as main predictors; rather, they were included to determine whether the predictive relationships between parental math involvement and children’s numeracy interest remained after accounting for potentially relevant background characteristics. Because the available evidence did not provide a consistent basis for directional expectations regarding the control variables, separate hypotheses were not formulated for them.

H1: Parental math involvement will be a significant positive predictor of children’s numeracy interest.

RQ1: Which domains of parental math involvement—Numbers and Operations, Geometry, Measurement, and Pattern—significantly predict children’s numeracy interest?

## Method

To address the two complementary gaps identified in the literature, the present research was organized into two quantitative studies. Study 1 employed a psychometric adaptation design to address the absence of a validated Turkish-language instrument for assessing children’s numeracy interest. The scale’s structural validity, internal consistency, and measurement invariance were therefore examined using EFA, CFA, reliability analyses, and multi-group CFA. Study 2 employed a cross-sectional correlational design to examine the underexplored association between parental math involvement and children’s numeracy interest in the Turkish early childhood context. Structural equation modeling was used to examine the associations of overall parental math involvement and its domains with children’s numeracy interest, both before and after accounting for relevant child and family characteristics. Parental math involvement and its domains were specified as predictor variables, whereas children’s numeracy interest was specified as the outcome variable. Given the cross-sectional design, the estimated relationships were interpreted as associations rather than evidence of causal effects.

### Participants

Study 1 addressed the Turkish adaptation of the Children’s Numeracy Interest Scale. Data were collected from volunteer parents of preschool children using convenience sampling. Data for both EFA and CFA were obtained through the same online survey. The sample consisted of 503 parents and was randomly divided into two independent and non-overlapping subsamples using a simple randomization procedure, with 200 participants assigned to the EFA sample and 303 assigned to the CFA sample. Most participants parents in Study 1 were female (92.6%), whereas 7.4% were male. Study 2 consisted of a separate sample of 394 parents, including 351 mothers (89.1%) and 43 fathers (10.9%). The inclusion criterion for both studies was having a preschool child aged 36–71 months. Study 1 and Study 2 were conducted with independent and non-overlapping samples; no parent participated in both studies. The sociodemographic characteristics of the samples are presented in Table 1. 

**Table 1:** Sociodemographic characteristics of the samples in Study 1 and Study 2

Characteristic	Study 1 (*N* = 503)		Study 2 (*N* = 394)	
	*N*	%	*N*	%
Parents’ gender				
Female	466	92.6	351	89.1
Male	37	7.4	43	10.9
Parents’ age				
18–35 years	309	61.4	222	56.3
36 years and older	194	38.6	172	43.7
Parents’ educational levels				
Primary school degree	34	6.8	20	5.1
Secondary school degree	76	15.1	34	8.6
High school degree	137	27.2	79	20.1
College/technical school degree	80	15.9	75	19.0
Bachelor’s degree	145	28.8	152	38.6
Master’s or doctorate degree	31	6.2	34	8.6
Children’s gender				
Girl	255	50.7	196	49.7
Boy	248	49.3	198	50.3
Children’s age				
36–41 months	36	7.2	38	9.6
42–47 months	32	6.4	30	7.6
48–53 months	105	20.9	88	22.3
54–59 months	62	12.3	53	13.5
60–65 months	126	25.0	91	23.1
66–71 months	142	28.2	94	23.9
Preschool attendance duration				
Less than 1 year	282	56.1	186	47.2
1–2 years	149	29.6	121	30.7
2 years or more	72	14.3	87	22.1
Number of siblings				
No siblings	164	32.6	141	35.8
One sibling	205	40.8	181	45.9
Two or more siblings	134	26.6	72	18.3

Sample-size adequacy was evaluated for both studies. For Study 1, it was assessed separately for the EFA and CFA subsamples. The EFA subsample (n = 200) exceeded Hair et al.’s [33] preferred minimum of 100 observations and the recommended minimum ratio of five observations per variable; it also exceeded the more acceptable 10:1 observation-to-variable ratio. For CFA, the six-item, one-factor measurement model was tested in an independent subsample of 303 participants. Given the standardized factor loadings reported in Table 3, which corresponded to some communalities below .45, the sample met Hair et al.’s [33] recommended minimum sample size of 300 for models with lower communalities. 

For Study 2, a priori power analysis was performed in R using the semPower package, which supports power analysis for structural equation modeling (SEM) applications, including regression and path models [34]. The analysis was based on the most complex structural regression model. Using a small-to-moderate expected standardized path coefficient of β = 0.20, an alpha level of 0.05, and a power of 0.90, the analysis showed that approximately 397 participants were required. Cohen [35] suggested that a minimum statistical power of 0.80 is generally adequate for behavioral research. Although the final SEM sample comprised 394 participants, three fewer than the estimated requirement of 397, this difference was considered minor, and the sample was therefore considered adequate for testing the proposed structural model.

### Measures

#### Demographic Information Form

The researcher-developed Demographic Information Form was used to obtain sociodemographic data from parents, including parental gender, age, and educational level. It also collected information about children’s gender, age, preschool attendance duration, and number of siblings. These variables were used to describe the study samples and, in Study 2, to control for demographic factors that could be associated with parental math involvement and children’s numeracy interest.

#### Early Math Questionnaire

Developed by Missall et al. [23], the Early Math Questionnaire comprises two components, namely Math Activities and Math Beliefs. It was designed to assess both parents’ engagement in mathematics-related activities with their preschool children and their beliefs concerning mathematics. Karakuş [36] adapted the questionnaire for use in the Turkish cultural context. The Math Activities Section was used in this study to examine parental math involvement both as an overall construct and in terms of its four domains. This section contains 36 items distributed across four domains: Numbers and Operations, Geometry, Measurement, and Pattern. Parents indicate how often each activity occurs using a five-point Likert-type scale scored from 0 (“not at all”) to 4 (“more than once a day”). In the original validation studies, this section demonstrated excellent internal consistency, with a Cronbach’s alpha coefficient of 0.96 [23, 37]. In the Turkish adaptation, reliability coefficients were reported as α = 0.97 and ω = 0.99. At the subscale level, the Numbers and Operations subscale showed α = 0.939 and ω = 0.937, the Geometry subscale showed α = 0.921 and ω = 0.923, the Measurement subscale showed α = 0.841 and ω = 0.831, and the Pattern subscale showed α = 0.923 and ω = 0.925 [36].

#### Children’s Numeracy Interest

The scale developed by Cheung et al. [1], was designed to assess children’s numeracy interest and consists of six number-related activities (e.g., participating in number games). Parents were asked to rate how much their children enjoyed each activity on a six-point scale ranging from 1 to 6 (1 = strongly dislike, 2 = moderately dislike, 3 = slightly dislike, 4 = slightly like, 5 = moderately like, 6 = strongly like). In the original validation study, principal component analysis supported a one-factor structure, with an eigenvalue of 3.51 explaining 58.45% of the total variance and factor loadings ranging from 0.69 to 0.82. Cronbach’s alpha for the scale was reported as 0.86 [1].

### Process

The study was conducted in accordance with the principles of the Declaration of Helsinki and approved by the Sinop University Human Research Ethics Committee (Date: 11 April 2025; Approval No: 2025/183). After obtaining permission from the developers of the Children’s Numeracy Interest Scale, the scale was adapted to the Turkish cultural context. The original version was independently translated into Turkish by the researcher and two specialists in preschool mathematics education, all of whom held doctoral degrees. These translations were then reviewed and combined into a single standardized Turkish form. Subsequently, two experts in English language education conducted the back-translation into English. To examine the clarity and content relevance of the Turkish version, three academics from different universities, all of whom held doctoral degrees in preschool mathematics education, reviewed the translated form. In the final step, the Turkish form was administered to five parents from the target population to evaluate whether the items were clear and understandable. The parents reported that all items were clear and understandable; therefore, no further revisions were required before the main data collection. This step served as a final comprehensibility check following forward translation, back-translation, and expert review, rather than as the sole procedure for establishing semantic and cultural equivalence. Wild et al. [38] recommended including 5–8 respondents in cognitive debriefing during instrument translation and cultural adaptation. Accordingly, the number of participants in the present study was consistent with the lower bound of this recommended range.

After ethical approval was obtained, parents were invited to participate in Study 1 with the assistance of school principals and teachers. The scale was administered online via Google Forms The online survey began with a Voluntary Participation Form, which informed participants about the purpose of the study, confidentiality, anonymity, the voluntary nature of participation, and their right to withdraw at any time. All participants provided informed consent before data collection began, and no personally identifiable information was requested. Participants completed the scale in approximately 5 min. The same ethical procedure was followed in Study 2. Participants were informed about the purpose of the study and asked to provide informed consent before completing the questionnaire. Data were collected in printed form through preschool education institutions. It took approximately 10 min for participants to complete the printed questionnaire.

### Data analysis

First, the data were screened for univariate and multivariate normality and for other assumptions relevant to the planned multivariate analyses. Univariate normality was assessed using skewness and kurtosis values, whereas multivariate normality was evaluated using Mardia’s multivariate kurtosis coefficient and its critical ratio. Because multivariate normality was not supported, bootstrap standard errors and bias-corrected 95% confidence intervals based on 5,000 resamples were used for the CFA factor loadings and structural path estimates. Descriptive statistics, including means, standard deviations, skewness, and kurtosis values, were calculated using IBM SPSS Statistics 27.0. The 95% confidence intervals for the means were calculated using the standard errors of the means and the corresponding *t*-distribution critical values. For both the baseline and conditional structural models, multicollinearity was assessed, where applicable, using variance inflation factor (VIF) and tolerance values for all predictor and control variables entered simultaneously in each model. VIF values below 10 and tolerance values above 0.10 were considered to indicate that extreme multicollinearity was unlikely [39]. Participants’ demographic characteristics were summarized using frequencies and percentages, and zero-order correlations were calculated to examine relationships among the study variables.

In the scale adaptation study, EFA was used to explore the underlying factor structure, whereas CFA was conducted to test the resulting model. EFA was performed in SPSS using maximum likelihood extraction. The number of factors to be retained was determined based on eigenvalues greater than 1 and inspection of the scree plot. Because these criteria supported a one-factor solution, no rotation was applied. CFA was conducted in AMOS version 22 using maximum likelihood estimation. To provide additional evidence of composite reliability and convergent validity, composite reliability and average variance extracted (AVE) were calculated using the standardized factor loadings and corresponding error variances obtained from the CFA. Composite reliability values of 0.70 or higher were considered indicative of adequate reliability, consistent with the general reliability criterion proposed by Nunnally and Bernstein [40], whereas AVE values of 0.50 or higher were considered indicative of adequate convergent validity [41]. Measurement invariance was subsequently tested using multi-group CFA.

Reliability was examined using Cronbach’s alpha and McDonald’s omega coefficients, with values of 0.70 or above considered indicative of acceptable internal consistency [40]. McDonald’s omega was calculated using the standardized factor loadings obtained from the CFA model.

Model fit was evaluated using multiple fit indices, including the chi-square-to-degrees-of-freedom ratio (χ²/df), comparative fit index (CFI), Tucker–Lewis index (TLI), root mean square error of approximation (RMSEA), and standardized root mean square residual (SRMR). Following Hu and Bentler [42], RMSEA values below 0.06, SRMR values below 0.08, and CFI and TLI values above 0.95 were considered indicative of good model fit. The magnitude of the standardized path coefficients (β) was interpreted according to Keith’s [43] criteria: coefficients ranging from 0.05 to 0.10 were considered small, those ranging from 0.11 to 0.25 moderate, and those greater than 0.25 large.

For the structural equation modeling analyses, the semPower package version 2.1.1 in R version 4.4.1 was used to determine the required sample size. The association between parental math involvement and children’s numeracy interest was examined using SEM. Models were estimated using maximum likelihood in AMOS Graphics version 22.0.

## Results

### Study 1

Study 1 evaluated the Turkish adaptation of the Children’s Numeracy Interest. Preliminary analyses showed that the skewness and kurtosis values for the total scale were − 0.811 and 0.127, respectively, indicating approximate univariate normality [39]. The suitability of the data for factor analysis was assessed using the Kaiser–Meyer–Olkin (KMO) coefficient and Bartlett’s test of sphericity. The KMO value was 0.884, and Bartlett’s test was significant, χ²(15) = 688.39, *p* < .001, indicating that the data were appropriate for factor analysis. The EFA, conducted using maximum likelihood extraction, supported a one-factor structure. This single factor explained 60.77% of the total variance. The complete factor matrix and item communalities are presented in Table 2.

**Table 2:** Factor loadings and communalities of the Turkish Children’s Numeracy Interest Scale

Item	Factor loading	Communality
Item 1	0.726	0.527
Item 2	0.765	0.585
Item 3	0.830	0.689
Item 4	0.831	0.690
Item 5	0.751	0.564
Item 6	0.769	0.591

Maximum likelihood extraction was used. Because a single factor was retained, no rotation was applied

As shown in Table 2, item factor loadings ranged from 0.726 to 0.831, and extraction communalities ranged from 0.527 to 0.690. Because a single factor was retained, cross-loadings were not applicable.

Before conducting the CFA, univariate and multivariate normality were examined in the CFA subsample (*n* = 303). Although no severe departures from univariate normality were observed at the item level, Mardia’s multivariate kurtosis results indicated that multivariate normality was not supported (multivariate kurtosis = 42.501, critical ratio = 37.753). Accordingly, bootstrap standard errors and bias-corrected 95% confidence intervals based on 5,000 resamples were used to evaluate the CFA parameter estimates.

The fit of the original one-factor structure to the Turkish sample was evaluated using CFA in AMOS 22. The model demonstrated good fit to the data, χ²/df = 1.881, CFI = 0.989, TLI = 0.982, RMSEA = 0.054, and SRMR = 0.026. These findings provided further support for the one-factor structure of the Turkish version of the scale. Item-level CFA parameter estimates, including unstandardized estimates, bootstrap standard errors, bias-corrected 95% bootstrap confidence intervals, p values, and standardized factor loadings, are presented in Table 3.

**Table 3:** CFA parameter estimates for the Turkish children’s numeracy interest scale

Item	Unstandardized estimate	Bootstrap SE	95% BC bootstrap CI	*p*	Standardized factor loading
Item 1	1.000	—	—	—	0.670
Item 2	1.111	0.132	[0.859, 1.371]	0.001	0.746
Item 3	1.207	0.108	[1.017, 1.442]	0.001	0.817
Item 4	1.172	0.121	[0.964, 1.443]	< 0.001	0.726
Item 5	1.122	0.136	[0.889, 1.417]	< 0.001	0.721
Item 6	1.049	0.137	[0.812, 1.354]	< 0.001	0.595

One unstandardized factor loading was fixed at 1.00 for model identification; therefore, its bootstrap standard error, confidence interval, and p value were not estimated

*Bootstrap SE * standard error based on 5,000 bootstrap resamples, *BC bootstrap CI*  bias-corrected 95% bootstrap confidence interval

As shown in Table 3, the standardized factor loadings ranged from 0.595 to 0.817. The bias-corrected 95% bootstrap confidence intervals for all freely estimated unstandardized factor loadings excluded zero, and all loadings were statistically significant (*p* ≤ .001). These findings provided further support for the one-factor structure of the Turkish version of the scale.

#### Reliability and convergent validity

In the total Study 1 sample (*N* = 503), the final Turkish version of the scale demonstrated high internal consistency, with a Cronbach’s alpha coefficient of 0.873. Split-half reliability was also assessed in the total sample. The Spearman–Brown coefficient was 0.852, and the Guttman split-half coefficient was 0.848, indicating good split-half reliability. Based on the CFA subsample (*n* = 303), the model-based McDonald’s omega and composite reliability coefficients were both 0.862, providing additional evidence of construct reliability. The AVE was 0.512, supporting convergent validity.

#### Measurement invariance

Measurement invariance across child gender was examined using multi-group CFA. Configural, metric, and scalar invariance models were tested sequentially, and the results are presented in Table 4. The configural model tested whether the same one-factor structure was supported across girls and boys. Metric invariance was examined by constraining factor loadings to be equal across groups, whereas scalar invariance was tested by additionally constraining item intercepts.

**Table 4:** Measurement invariance across children’s gender

Model	χ2/df	CFI	TLI	RMSEA	SRMR	ΔCFI	ΔRMSEA	ΔSRMR
Configural	2.148	0.985	0.974	0.048	0.029	—	—	—
Metric	1.745	0.987	0.983	0.039	0.030	.002	−.009	.001
Scalar	1.570	0.988	0.987	0.034	0.030	.001	−.005	.000

Δ values represent changes compared with the previous model

Measurement invariance was examined across child gender because both gender groups had adequate sample sizes. Invariance was not examined across child age or parent education because these variables would have required six-group comparisons involving several small groups and substantially unequal group sizes. Insufficient sample sizes may be associated with biased parameter estimates and a greater risk of model estimation problems [44], whereas severe imbalances in group sizes may reduce the ability to detect measurement noninvariance [45]. Therefore, these comparisons were not considered sufficiently robust.

Changes in model fit, presented in Table 4, were evaluated using the cutoff criteria recommended by Chen [46]. Metric invariance was considered supported when the decrease in CFI (ΔCFI) did not exceed 0.010 and the increases in RMSEA (ΔRMSEA) and SRMR (ΔSRMR) did not exceed 0.015 and 0.030, respectively. For scalar invariance, the corresponding thresholds were 0.010 for the decrease in CFI and 0.015 and 0.010 for the increases in RMSEA and SRMR, respectively. As shown in Table 4, the changes in CFI, RMSEA, and SRMR remained within these recommended limits. These findings supported both metric and scalar invariance across child gender. Metric invariance indicates that the items measured the latent construct comparably for girls and boys, whereas scalar invariance supports meaningful comparisons of latent mean levels between the two groups.

### Study 2

#### Measurement model and reliability analysis

Before testing the structural model, CFA was conducted to examine whether the factor structures of the scales were supported in the present sample. Because multivariate normality was not supported, bootstrap standard errors and bias-corrected 95% confidence intervals based on 5,000 resamples were used to evaluate the CFA parameter estimates.

As shown in Table 5, the CFA results indicated that both Parental math Involvement and Children’s Numeracy Interest showed acceptable model fit in the present sample. All freely estimated factor loadings were statistically significant, and their bias-corrected 95% bootstrap confidence intervals excluded zero. The standardized factor loadings are presented in Table 6.

**Table 5:** CFA results for the scales

Scales	χ2/df	CFI	TLI	RMSEA	SRMR
Parental Math Involvement Children’s Numeracy Interest	2.614 2.765	0.910 0.991	0.901 0.976	0.064 0.067	0.057 0.020

**Table 6:** Standardized factor loadings for the five-factor measurement model

Factor	Item	Standardized loading (λ)
Numbers and Operations	PMI1	.529
	PMI2	.533
	PMI3	.512
	PMI4	.631
	PMI5	.684
	PMI6	.618
	PMI7	.604
	PMI8	.698
	PMI9	.708
	PMI10	.705
	PMI11	.631
	PMI12	.747
	PMI13	.783
	PMI14	.783
	PMI15	.741
	PMI16	.695
	PMI17	.703
	PMI18	.743
	PMI19	.739
	PMI29	.740
Geometry	PMI20	.727
	PMI21	.785
	PMI22	.760
	PMI23	.766
	PMI24	.791
	PMI25	.800
	PMI26	.714
	PMI27	.746
	PMI28	.691
Measurement	PMI30	.851
	PMI31	.869
	PMI32	.577
	PMI33	.620
Pattern	PMI34	.862
	PMI35	.959
	PMI36	.910
Children’s Numeracy Interest	CNI1	.684
	CNI2	.659
	CNI3	.818
	CNI4	.802
	CNI5	.735
	CNI6	.722

*PMI* Parental Math Involvement, *CNI* Children’s Numeracy Interest

As shown in Table 6, all indicators loaded significantly on their intended factors. Standardized factor loadings ranged from 0.512 to 0.783 for Numbers and Operations, from 0.691 to 0.800 for Geometry, from 0.577 to 0.869 for Measurement, from 0.862 to 0.959 for Pattern, and from 0.659 to 0.818 for Children’s Numeracy Interest. The correlations among the latent factors are presented in Table 7.

**Table 7:** Correlations among latent factors of the five-factor measurement model

Latent factor	1	2	3	4	5
1. Numbers and Operations	-				
2. Geometry	0.834***	-			
3. Measurement	0.757***	0.874***	-		
4. Pattern	0.693***	0.681***	0.756***	-	
5. Children’s Numeracy Interest	0.377***	0.239***	0.279***	0.282***	-

All latent-factor correlations were statistically significant

***p < .001

As presented in Table 7, the correlations among the latent factors ranged from 0.239 to 0.874, and all correlations were statistically significant at *p* < .001.

As shown in Table 8, all scales and subscales demonstrated high internal consistency in the Study 2 sample, with Cronbach’s alpha coefficients ranging from 0.836 to 0.967 and McDonald’s omega coefficients ranging from 0.825 to 0.972.

**Table 8:** Internal consistency reliability coefficients of the scales and subscales

Scale/Subscale	Cronbach’s alpha coefficient (α)	McDonald’s omega coefficient (ω)
Children’s Numeracy Interest	0.872	0.875
Parental Math involvement	0.967	0.972
Numbers and Operations	0.945	0.945
Geometry	0.925	0.922
Measurement	0.836	0.825
Pattern	0.934	0.936

#### Descriptive statistics and correlations between variables

Table 9 presents the means, standard deviations (SDs), 95% confidence intervals (CIs), skewness, and kurtosis values for the study variables.

**Table 9:** Descriptive statistics and 95% confidence intervals for the study variables

Variables	M (SD)	95% CI for M	Skewness	Kurtosis
Children’s numeracy interest	31.38 (4.19)	[30.96, 31.79]	− 0.78	− 0.16
Parental math involvement	126.82 (34.36)	[123.42, 130.23]	− 0.24	− 0.87
Numbers and Operations	68.54 (19.77)	[66.58, 70.49]	− 0.17	− 0.97
Geometry	33.68 (9.51)	[32.74, 34.62]	− 0.54	− 0.78
Measurement	14.01 (4.65)	[13.54, 14.47]	− 0.29	− 0.98
Pattern	10.60 (3.96)	[10.21, 11.00]	− 0.50	− 0.99

As presented in Table 9, skewness values ranged from − 0.78 to − 0.17, and kurtosis values ranged from − 0.99 to − 0.16. These values indicated that the variables did not substantially deviate from univariate normality [39]. The zero-order correlations among the main study variables and the control variables are presented in Table 10.

**Table 10:** Zero-order correlations among the main study and control variables

	Variables	1	2	3	4	5	6	7	8	9	10	11	12	13
1	Parents’ gender	-												
2.	Parents’ age	0.17**	-											
3.	Parents’ educational levels	0.11*	0.07	-										
4.	Children’s gender	0.04	0.01	0.06	-									
5.	Children’s age	0.01	0.06	− 0.26**	− 0.01	-								
6.	Preschool attendance duration	0.01	0.16**	0.23**	− 0.01	0.37**	-							
7.	Number of siblings	0.03	0.23**	− 0.29**	− 0.12*	0.17**	− 0.10	-						
8.	Numbers and Operations	− 0.07	− 0.02	0.07	0.04	0.09	0.06	− 0.04	-					
9.	Geometry	− 0.06	− 0.00	0.11*	0.01	− 0.06	0.02	− 0.07	0.77**	-				
10.	Measurement	− 0.03	0.04	0.06	0.10*	0.02	0.03	0.00	0.71**	0.74**	-			
11.	Pattern	− 0.01	0.08	0.05	− 0.02	0.06	0.05	0.04	0.66**	0.65**	0.71**	-		
12.	Parental math involvement	− 0.06	0.01	0.08	0.03	0.04	0.05	− 0.04	0.96**	0.89**	0.83**	0.77**	-	
13.	Children’s numeracy interest	− 0.03	0.09	0.00	− 0.02	0.22**	0.16**	0.08	0.34**	0.22**	0.25**	0.25**	0.32**	-

* *p* < .05; ** *p* < .01

As shown in Table 10, parental math involvement was moderately and positively correlated with children’s numeracy interest (*r* = .32, *p* < .01). Children’s numeracy interest was also moderately and positively correlated with Numbers and Operations (*r* = .34, *p* < .01), and weakly and positively correlated with Geometry (*r* = .22, *p* < .01), Measurement (*r* = .25, *p* < .01), and Pattern (*r* = .25, *p* < .01). Although these associations were statistically significant, their weak-to-moderate magnitudes suggest that parental math involvement is related to children’s numeracy interest and that other factors may also play an important role. Some demographic characteristics were also significantly associated with the main study variables.

The four domains of parental math involvement were strongly correlated with one another (*r* = .65–0.77), indicating that they are closely related aspects of overall parental math involvement. The particularly high correlation between overall parental math involvement and Numbers and Operations (*r* = .96) should be interpreted partly as a part–whole association because the overall score was calculated from the items of all four domains, and Numbers and Operations contained the largest number of items. To avoid this overlap, the overall score and the four domain scores were examined in separate structural models rather than entered simultaneously. Accordingly, each domain-specific coefficient represents the unique association of that domain with children’s numeracy interest after controlling for the other domains.

#### Structural model results

##### Model specifications

Four models were tested: two baseline models (Model 1 and Model 2) and two conditional models (Model 3 and Model 4).

In the baseline models, parental math involvement was examined as a predictor of children’s numeracy interest without including control variables. Model 1 specified overall parental math involvement as a predictor of children’s numeracy interest. Model 2 examined whether the domains of parental math involvement—Numbers and Operations, Geometry, Measurement, and Pattern—predicted children’s numeracy interest.

In the conditional models, theoretically relevant control variables were added to assess whether these associations remained after accounting for selected child and family characteristics. The control variables were selected based on prior research, their theoretical relevance to parental math involvement and children’s numeracy interest, and their availability in the dataset. Accordingly, child age, child gender, preschool attendance duration, parental education, and number of siblings were included in the structural models. Family socioeconomic status and parental attitudes toward mathematics were not directly assessed and therefore could not be included. Direct paths were specified from these control variables to children’s numeracy interest, and covariances were specified between the parental math involvement variables and the control variables. Model 3 extended Model 1 by including the five control variables. Model 4 extended Model 2 by including the same control variables while simultaneously estimating the associations of the four parental math involvement domains with children’s numeracy interest.

##### Assessment of multivariate normality

Mardia’s multivariate kurtosis results indicated that multivariate normality was not supported in the baseline models (Models 1 and 2: multivariate kurtosis = 29.352, critical ratio = 18.804) and in the conditional models (Models 3 and 4: multivariate kurtosis = 23.264, critical ratio = 10.224). Accordingly, bootstrap standard errors and bias-corrected 95% confidence intervals based on 5,000 resamples were used for all structural path estimates.

##### Multicollinearity diagnostics

Multicollinearity was assessed for all structural models containing multiple predictors. In Model 2, VIF values ranged from 2.288 to 3.054, and tolerance values ranged from 0.327 to 0.437. In Model 3, VIF values ranged from 1.013 to 1.383, and tolerance values ranged from 0.723 to 0.988. In Model 4, VIF values ranged from 1.055 to 3.212, and tolerance values ranged from 0.311 to 0.948. Thus, all VIF values were below the commonly suggested threshold of 10, and all tolerance values were above 0.10, indicating that multicollinearity was not a serious concern [39].

##### Goodness of fit

Before examining the structural path estimates, the fit of the baseline and conditional models was evaluated using standard goodness-of-fit indices.

The indices reported in Table 11 indicated acceptable fit for all tested models according to the criteria of Hu and Bentler [42].

**Table 11:** Fit indices for the structural models

Model	χ2/df	CFI	TLI	RMSEA	SRMR
Model 1 (Baseline model)	2.841	0.973	0.962	0.068	0.038
Model 2 (Baseline model)	2.053	0.987	0.978	0.052	0.028
Model 3 (Conditional model)	2.091	0.967	0.952	0.053	0.035
Model 4 (Conditional model)	1.550	0.988	0.976	0.037	0.026

##### Parameter estimates for the baseline models

**Fig. 1:**
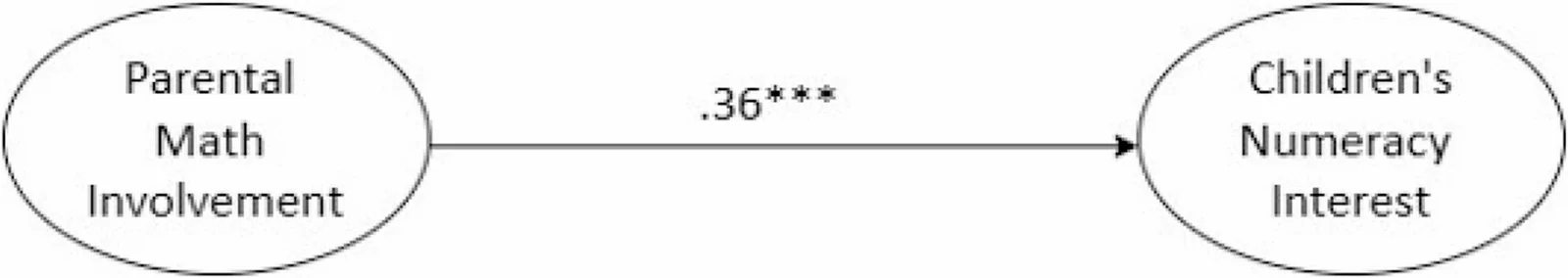
Model 1 (Baseline model): Parental math involvement as a predictor of children’s numeracy interest. Note. Standardized path coefficient is reported. *** *p *< .001

**Fig. 2:**
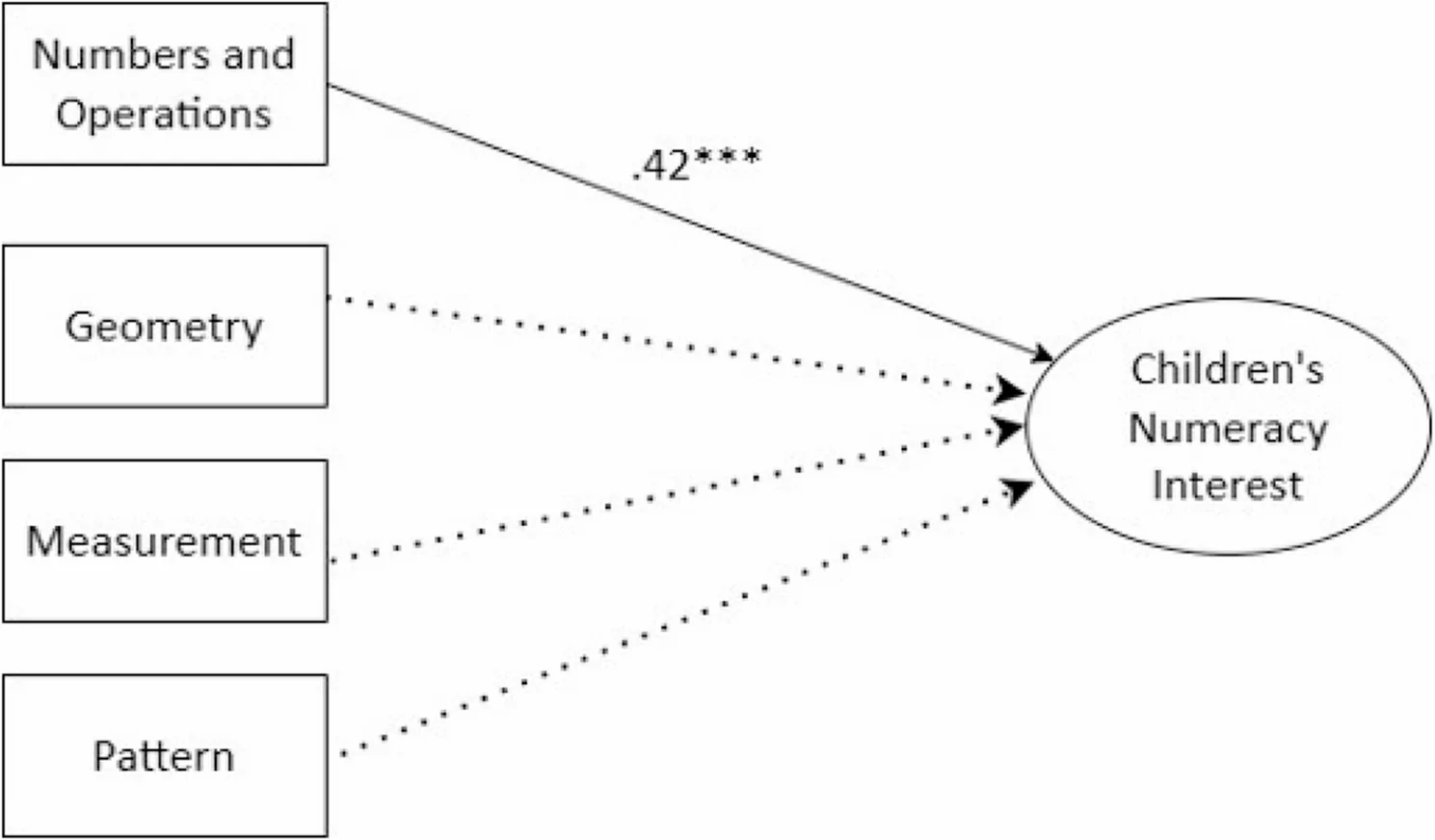
Model 2 (Baseline model): Parental math involvement domains as predictors of children’s numeracy interest. Note. Standardized path coefficients are reported. Only significant path coefficients are displayed in the figure; nonsignificant paths are shown with dotted lines for clarity. The four parental math involvement domains were allowed to covary, but covariances are not displayed in the figure for clarity. ***p < .001

Figures 1 and 2 present the results of the baseline models. Model 1 examined whether overall parental math involvement predicted children’s numeracy interest, as shown in Fig. 1.

As shown in Fig. 1, parental math involvement was a significant positive predictor of children’s numeracy interest (β = 0.36, *p* < .001). Model 1 accounted for approximately 12.7% of the variance in children’s numeracy interest. According to Keith’s [43] criteria for interpreting standardized path coefficients, this path coefficient was considered large.

Then, Model 2 examined whether the specific parental math involvement domains—Numbers and Operations, Geometry, Measurement, and Pattern—predicted children’s numeracy interest, as shown in Fig. 2.

As shown in Fig. 2, when the four parental math involvement domains were entered simultaneously, only the Numbers and Operations domain was a significant positive predictor predictor of children’s numeracy interest (β = 0.42, *p* < .001). Geometry, Measurement, and Pattern domains were not significant predictors. Model 2 accounted for approximately 15.6% of the variance in children’s numeracy interest. Based on Keith’s [43] criteria for interpreting standardized path coefficients, the path coefficient for Numbers and Operations was considered large.

##### Parameter estimates for the conditional models

**Fig. 3:**
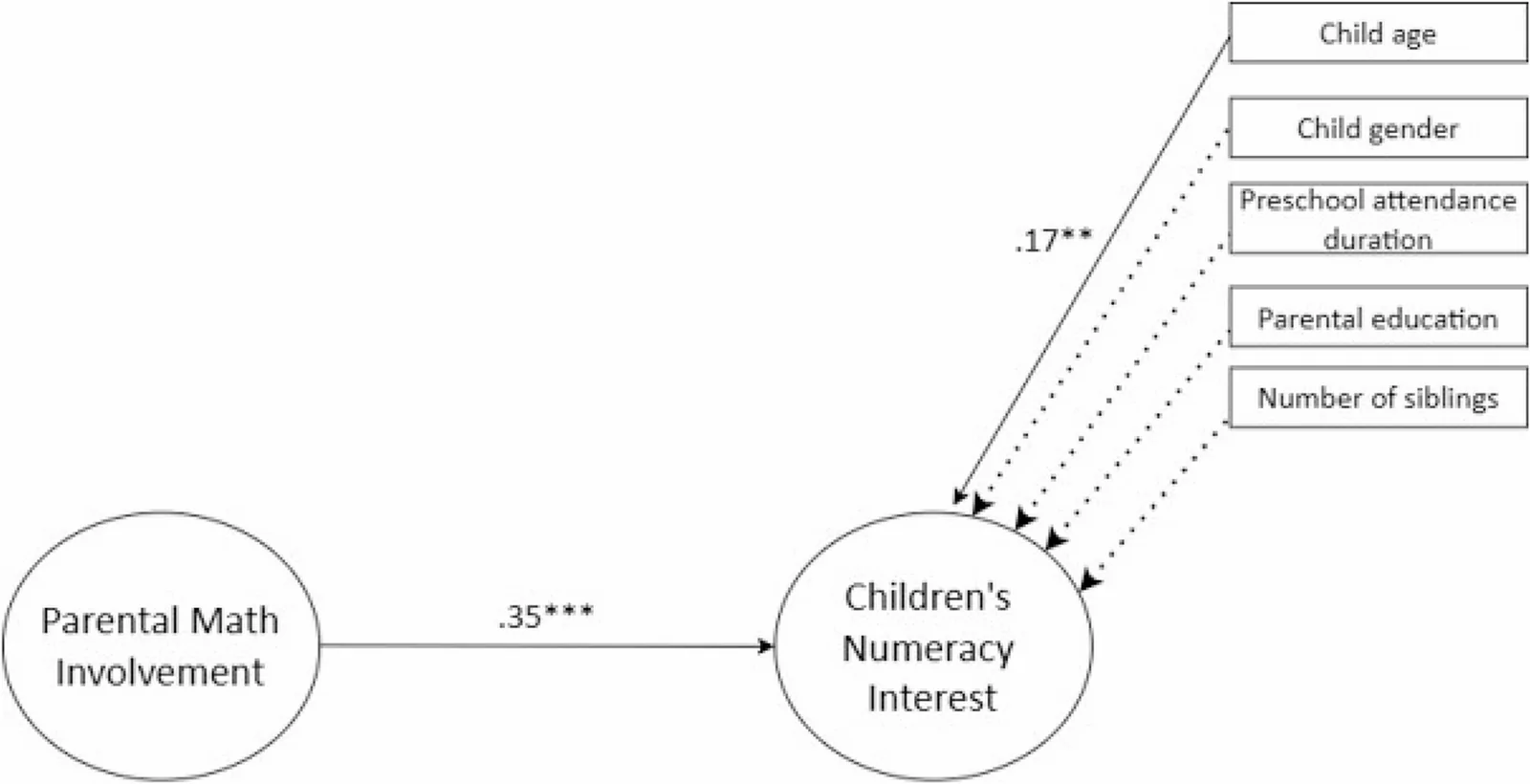
Model 3 (Conditional model): Parental math involvement as a predictor of children’s numeracy interest after controlling for child and family characteristics. Note. Standardized path coefficients are reported. Only significant path coefficients are displayed in the figure; nonsignificant paths are shown with dotted lines for clarity. The predictor and control variables were allowed to covary, but covariances are not displayed for clarity. **p < .01, ***p < .001

**Fig. 4:**
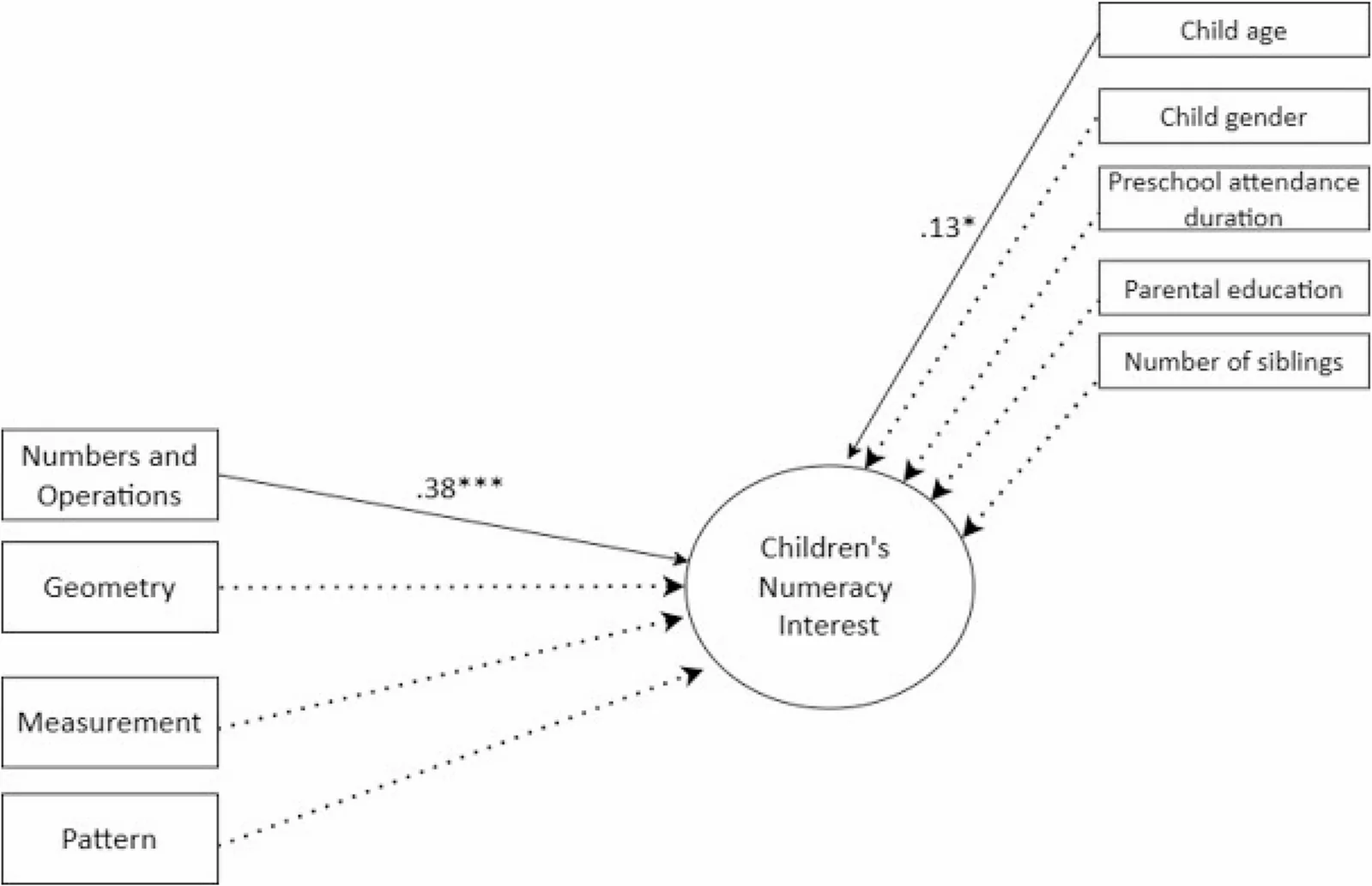
Model 4 (Conditional model): Parental math involvement domains as predictors of children’s numeracy interest after controlling for child and family characteristics. Note. Standardized path coefficients are reported. Only significant path coefficients are displayed in the figure; nonsignificant paths are shown with dotted lines for clarity. The parental math involvement domains and control variables were allowed to covary, but covariances are not displayed in the figure for clarity. *p < .05, ***p < .001

The results of the conditional models are presented in Figs. 3 and 4. Model 3 examined whether overall parental math involvement predicted children’s numeracy interest after controlling for child and family characteristics.

As shown in Fig. 3, parental math involvement remained a significant positive predictor of children’s numeracy interest after controlling for child and family characteristics (β = 0.35, *p* < .001). Among the control variables, only child age significantly predicted children’s numeracy interest (β = 0.17, *p* < .01). Child gender, preschool attendance duration, parental education, and number of siblings were not significant predictors. Model 3 accounted for approximately 18.1% of the variance in children’s numeracy interest. According to Keith’s [43] criteria for interpreting standardized path coefficients, the path coefficient for parental math involvement was considered large.

Then, Model 4 tested whether the domains of parental math involvement predicted children’s numeracy interest after including the same control variables.

As shown in Fig. 4, when the four parental math involvement domains and control variables were entered simultaneously, the Numbers and Operations domain remained a significant positive predictor of children’s numeracy interest (β = 0.38, *p* < .001). Geometry, Measurement, and Pattern domains were not significant predictors. Among the control variables, only child age significantly predicted children’s numeracy interest (β = 0.13, *p* < .05). Child gender, preschool attendance duration, parental education, and number of siblings were not significant predictors. Model 4 accounted for approximately 19.8% of the variance in children’s numeracy interest. Based on Keith’s [43] criteria for interpreting standardized path coefficients, the path coefficient for Numbers and Operations was considered large.

Table 12 summarizes the structural path estimates for overall parental math involvement and its domains, including parameter estimates, bootstrap standard errors, bias-corrected 95% bootstrap confidence intervals, and R² values.

**Table 12:** Parameter estimates and bootstrap confidence intervals for the baseline and conditional models

Model	Path	B	Bootstrap SE	95% BC bootstrap CI	β	*p*	*R*²
Model 1	Parental math involvement → Children’s numeracy interest	.052	.009	[0.034, 0.070]	.356	< 0.001	.127
Model 2	Numbers and Operations → Children’s numeracy interest Geometry → Children’s numeracy interest Measurement → Children’s numeracy interest Pattern → Children’s numeracy interest	.009 −0.008 .007 .007	.002 .004 .008 .009	[0.006, 0.014] [− 0.016, 0.000] [− 0.010, 0.023] [− 0.010, 0.025]	.418 −0.159 .070 .058	< 0.001 .064 .425 .453	.156
Model 3	Parental math involvement → Children’s numeracy interest Child age → Children’s numeracy interest Child gender → Children’s numeracy interest Preschool attendance duration → Children’s numeracy interest Parental education → Children’s numeracy interest Number of siblings → Children’s numeracy interest	.051 .046 −0.012 .054 .005 .041	.009 .017 .047 .034 .019 .036	[0.033, 0.069] [0.014, 0.081] [− 0.107, 0.080] [− 0.013, 0.120] [− 0.031, 0.042] [− 0.031, 0.109]	.348 .166 −0.013 .095 .016 .065	< 0.001 .005 .789 .103 .753 .250	.181
Model 4	Numbers and Operations → Children’s numeracy interest Geometry → Children’s numeracy interest Measurement → Children’s numeracy interest Pattern → Children’s numeracy interest Child age → Children’s numeracy interest Child gender → Children’s numeracy interest Preschool attendance duration → Children’s numeracy interest Parental education → Children’s numeracy interest Number of siblings → Children’s numeracy interest	.009 −0.005 .007 .003 .037 −0.017 .055 .007 .043	.002 .004 .009 .009 .017 .049 .033 .019 .035	[0.004, 0.013] [− 0.014, 0.003] [− 0.010, 0.024] [− 0.013, 0.022] [0.005, 0.072] [− 0.117, 0.075] [− 0.008, 0.122] [− 0.029, 0.043] [− 0.028, 0.108]	.375 −0.104 .074 .030 .134 −0.018 .098 .019 .068	< 0.001 .226 .418 .691 .023 .734 .091 .685 .227	.198

B = unstandardized maximum likelihood estimate; Bootstrap SE = standard error based on 5,000 bootstrap resamples; BC bootstrap CI = bias-corrected 95% bootstrap confidence interval for B; β = standardized coefficient; p = bootstrap p value; R² = proportion of variance explained in children’s numeracy interest

## Discussion

The validation of the Turkish version of the Children’s Numeracy Interest Scale addresses an important measurement gap in early childhood mathematics research in Türkiye. The supported one-factor structure suggests that a brief parent-report scale can assess children’s interest in number-related activities as a single construct. Moreover, evidence of metric and scalar invariance across child gender indicates that the scale measures numeracy interest comparably for girls and boys and supports meaningful comparisons between these groups. Thus, the Turkish version provides a psychometrically supported instrument that can facilitate further research on the development, correlates, and educational significance of numeracy interest among among preschool children in Türkiye.

Study 2 showed that greater parental math involvement was associated with higher levels of children’s numeracy interest. However, the baseline model accounted for 12.7% of the variance in numeracy interest, indicating relatively modest explanatory power. Therefore, parental math involvement should be regarded as one of several factors associated with children’s numeracy interest rather than as a dominant predictor. The considerable amount of unexplained variance further suggests that other child, family, and contextual factors may contribute to individual differences in numeracy interest. Moreover, given the cross-sectional design and the concurrent assessment of the variables, temporal precedence and causal direction could not be established [47]. Parental math involvement may provide children with more opportunities to engage in numeracy-related activities; however, children with greater numeracy interest may also encourage more frequent mathematical involvement from their parents. Thus, the association may be bidirectional rather than unidirectional. This finding is consistent with previous research indicating the potential relevance of home-based mathematical experiences to children’s developing interest in numeracy. Cheung et al. [1], for example, found that young Filipino children’s numeracy interest was associated with their parents’ practices and attitudes. Longitudinal evidence from Hong Kong similarly showed that mother–child home numeracy activities were associated with preschool children’s numeracy interest [18]. Hao et al. [30] further reported that home numeracy activities mediated the association between parental attitudes toward children’s numeracy learning and children’s numeracy interest. Cheung et al. [29] also found that both the frequency of home numeracy activities and parents’ use of indirect numeracy teaching strategies were positively associated with children’s numeracy interest. More recently, Chen et al. [28] showed that home numeracy activities and parental involvement in school mediated the association between parental attitudes and numeracy interest among Chinese kindergarten children. Overall, the available evidence suggests that both the frequency and nature of parents’ involvement in children’s mathematical learning may be relevant to children’s numeracy interest. This association can also be interpreted in light of the four-phase model of interest development and ecological systems theory. Within the family microsystem [15], repeated parent–child mathematical activities may provide opportunities for situational interest to be triggered and maintained over time [14]. Continued participation in these experiences may be associated with the gradual development of more sustained interest in numeracy-related activities. The present study extends this line of research to preschool children in Türkiye, where, to the best of my knowledge, the relationship between parental math involvement and children’s numeracy interest has not previously been examined.

The domain-level analyses provided further insight into this relationship. Although all domains of parental math involvement were positively correlated with children’s numeracy interest, only involvement in Numbers and Operations remained uniquely associated with numeracy interest when the domains were examined simultaneously. This association remained significant after accounting for the child and family characteristics included in the model. This distinction is important because the association of each domain with numeracy interest was estimated while accounting for its overlap with the other domains. Given the relatively strong correlations among the parental math involvement domains, the nonsignificant findings for Geometry, Measurement, and Pattern do not mean that these domains were unrelated to numeracy interest. Rather, they suggest that these domains did not make an additional unique contribution once their shared variance with Numbers and Operations and with the other domains was taken into account. This pattern is consistent with evidence that specific components of the home mathematics environment may not contribute equally once their shared variance is considered [4]. Although Ouyang et al. [18] examined different types of number-related activities rather than distinct mathematical domains, they similarly found that only number-skill activities made a unique contribution to children’s numeracy interest when all activity types were considered together. This finding supports examining parental math involvement as a multidimensional construct because its domains may differ in their unique associations with children’s numeracy interest when their shared variance is taken into account.

The correspondence between the content of the parental math involvement measure and the outcome measure may be particularly important in explaining this finding. Parental math involvement included activities in Numbers and Operations, Geometry, Measurement, and Pattern, whereas the numeracy interest scale primarily assessed children’s interest in number-focused experiences, such as identifying numbers, counting, and writing numbers. Activities in the Numbers and Operations domain therefore corresponded more closely to the content assessed by the outcome measure.From the perspective of the four-phase model of interest development, repeated engagement in experiences that closely correspond to a particular content area may help trigger and maintain situational interest in that area [14]. Accordingly, number-focused parental activities may have been more directly reflected in scores on the numeracy interest scale than activities involving geometry, measurement, or pattern. The unique association of Numbers and Operations may therefore reflect, at least partly, this content correspondence rather than indicating that this domain is inherently more important than the other mathematical domains. The nonsignificant findings for Geometry, Measurement, and Pattern should therefore not be interpreted as evidence that activities in these domains are unimportant, as the scale did not directly assess children’s interest in these areas. This possibility should be examined in future research using domain-specific measures of mathematical interest.

Another possible explanation is that number-focused activities may be more visible or easier for parents to recognize and report than mathematical experiences embedded in geometry, measurement, or pattern activities. Moreover, because both parental involvement and children’s interest were reported by parents, the conceptual similarity between number-focused items across the two measures may have strengthened the observed association. The cross-sectional design also leaves open the possibility of a reciprocal relationship: parental involvement in Numbers and Operations may support children’s numeracy interest, but children who are already interested in numbers may also encourage their parents to engage them more frequently in number-related activities.

Among the child and family characteristics examined, only child age was uniquely associated with children’s numeracy interest. Older preschool children were rated as showing greater interest in numeracy-related activities after accounting for parental math involvement and the other background characteristics. One possible explanation is that older children have had more opportunities to encounter and participate in number-related activities involving counting, number games, and simple operations. From the perspective of the four-phase model of interest development, repeated and supported experiences may help maintain situational interest and contribute to the development of a more enduring interest over time [14]. Because interest can develop through repeated engagement, older children may have had more opportunities to encounter numerical content, allowing situational interest to be triggered and sustained over time. This interpretation is consistent with evidence that parents of older preschool children engage in home numeracy activities more frequently and that more advanced home numeracy activities are associated with older children’s numeracy performance [48]. Greater exposure to such activities may, in turn, provide additional opportunities for children’s numeracy interest to develop.

In contrast, child gender, duration of preschool attendance, parental education, and number of siblings were not uniquely associated with numeracy interest in the structural models. The nonsignificant unique association of preschool attendance duration may partly reflect its overlap with child age. This finding is broadly consistent with Cheung et al. [1], who found that children’s numeracy interest was associated with parental practices and attitudes, whereas the demographic variables included in their model did not make significant contributions. The present findings similarly suggest that children’s everyday mathematical experiences and interactions with parents may be more closely related to numeracy interest than the child and family background characteristics examined.

From an ecological perspective, children’s development is shaped by their experiences and interactions within immediate environments, as well as by the broader contexts surrounding these environments [15]. Accordingly, child gender, parental education, and number of siblings may be associated with numeracy interest partly through more immediate experiences, such as the frequency and quality of parent–child mathematical interactions. This may help explain why these control variables did not account for additional unique variance once parental math involvement was included in the model. With respect to child gender, Kersey et al. [49] found no gender differences in early numerical and mathematical abilities. Although their study focused on ability rather than interest, this finding is broadly consistent with the absence of a significant association between child gender and numeracy interest in the present study. However, the absence of significant associations should not be interpreted as evidence that these characteristics are unimportant for early mathematical development. Rather, they may be more relevant to children’s mathematical competence, access to learning opportunities, or the quality and frequency of the mathematical experiences available to them.

The findings also have practical implications for early childhood education, although they should not be interpreted as evidence of causal effects. Early childhood educators could encourage parents to integrate simple, developmentally appropriate number-related activities into everyday family routines. Experiences such as counting, comparing quantities, and discussing numbers may provide opportunities for children to engage with numeracy in meaningful everyday contexts. However, given the relatively small proportion of variance explained, the cross-sectional design and reliance on parent-reported data, these practical implications should be interpreted cautiously and examined further through longitudinal and intervention studies.

## Limitations and future directions

Despite the important contributions of the present study, several limitations should be acknowledged. First, the cross-sectional design precludes conclusions about the direction of the relationship between parental math involvement and children’s numeracy interest. Although parental math involvement accounted for unique variance in numeracy interest, this statistical association should not be interpreted as evidence that greater parental involvement leads to greater interest. The reverse direction is also possible, such that children who display greater numeracy interest may elicit more mathematical involvement from their parents. Longitudinal and cross-lagged studies could clarify how parental math involvement and children’s numeracy interest are related over time, whereas intervention studies designed to enhance parents’ engagement in home-based mathematical activities could provide stronger evidence regarding potential causal effects.

Second, the parent sample was predominantly female (89.1%), which limits the representativeness of the findings, particularly with respect to fathers. Consequently, the findings may primarily reflect mothers’ perceptions and parental math involvement practices, and caution is warranted when generalizing them to parental math involvement more broadly. Future studies should recruit more gender-balanced samples of parents and examine whether parental math involvement and its association with children’s numeracy interest differ according to parent gender.

Third, both parental math involvement and children’s numeracy interest were assessed using parent reports. The observed associations may therefore have been affected by social desirability, shared reporter effects, or common method variance. Although parent-report measures provide valuable information about home-based mathematical activities, they may not fully capture the quality, context, and interactive nature of parent–child mathematical experiences. Future studies could address this limitation through a multimethod design that integrates parent reports, naturalistic observations, teacher reports, and direct assessments of children.

Fourth, because the Turkish version of the Children’s Numeracy Interest Scale was administered only once in Study 1, its test–retest reliability could not be assessed. Future studies should evaluate the stability of the scale scores across repeated administrations.

Fifth, measurement invariance was examined only across child gender. Future studies with larger samples within each child age and parent education category should examine the measurement invariance of the scale across these characteristics.

Sixth, the numeracy interest scale primarily assessed children’s interest in number-focused activities. Accordingly, the findings should not be extended to children’s interest in other mathematical domains, and the number-focused content of the scale may partly explain the unique association found for Numbers and Operations. Future studies could develop or employ measures that assess children’s interest separately across Numbers and Operations, Geometry, Measurement, and Pattern. Such measures would allow the domain-specific relationships between parental involvement and children’s mathematical interest to be examined more directly.

Finally, although the analyses included several child and family characteristics, other potentially relevant factors were not assessed. In particular, family socioeconomic status was not directly assessed. Although parental education was included as an indicator of family background, it represents only one dimension of socioeconomic status and should not be interpreted as a comprehensive measure of families’ socioeconomic conditions. Broader indicators of socioeconomic status, such as household income and parental occupation, as well as parental mathematics beliefs and attitudes, the availability of home numeracy resources, and the quality of parent–child mathematical interactions may be associated with both parental math involvement and children’s numeracy interest. Incorporating these factors into future research could provide a more comprehensive understanding of the relationship between the home numeracy environment and children’s numeracy interest.

## Conclusion

This study provides initial evidence supporting the use of the Children’s Numeracy Interest Scale in Türkiye and contributes to research on the relationship between parental math involvement and preschool children’s numeracy interest. The Turkish version demonstrated a single-factor structure, satisfactory reliability, and metric and scalar invariance across child gender. Greater parental math involvement was positively associated with children’s numeracy interest.

When the domains of parental math involvement were considered simultaneously, only Numbers and Operations showed a unique positive association with numeracy interest. However, this domain-specific finding should be interpreted cautiously because the outcome measure primarily assessed children’s interest in number-related activities. Moreover, the cross-sectional design and parent-reported data preclude causal conclusions. Nevertheless, the findings suggest that developmentally appropriate number-related experiences embedded in everyday family routines may be relevant to children’s numeracy interest. The study also provides a psychometrically supported instrument for examining numeracy interest in Türkiye and contributes to a broader understanding of the affective dimension of early mathematical development.

## Data Availability

The datasets generated and/or analysed during the current study are not publicly available due to ethical and confidentiality considerations but are available from the corresponding author on reasonable request.

## Abbreviations

AMOS: Analysis of Moment Structures
AVE: Average variance extracted
BC: Bias-corrected
CFA: Confirmatory factor analysis
CFI: Comparative fit index
CI: Confidence interval
EFA: Exploratory factor analysis
KMO: Kaiser–Meyer–Olkin
RMSEA: Root mean square error of approximation
SD: Standard deviation
SE: Standard error
SEM: Structural equation modeling
SPSS: Statistical Package for the Social Sciences
SRMR: Standardized root mean square residual
TLI: Tucker–Lewis index
VIF: Variance inflation factor
χ²/df: Chi-square-to-degrees-of-freedom ratio
